# Dynamic *Trans* Interactions in Yeast Chromosomes

**DOI:** 10.1371/journal.pone.0075895

**Published:** 2013-09-30

**Authors:** Ekaterina V. Mirkin, Frederick S. Chang, Nancy Kleckner

**Affiliations:** Department of Molecular and Cellular Biology, Harvard University, Cambridge, Massachusetts, United States of America; Oklahoma Medical Research Foundation, United States of America

## Abstract

Three-dimensional organization of the genome is important for regulation of gene expression and maintenance of genomic stability. It also defines, and is defined by, contacts between different chromosomal loci. Interactions between loci positioned on different chromosomes, i.e. *“trans”* interactions are one type of such contacts. Here, we describe a case of inducible *trans* interaction in chromosomes of the budding yeast *S. cerevisiae*. Special DNA sequences, inserted in two ectopic chromosomal loci positioned *in trans*, pair with one another in an inducible manner. The spatial proximity diagnostic of pairing is observable by both chromosome capture analysis (3C) and epifluorescence microscopy in whole cells. Protein synthesis *de novo* appears to be required for this process. The three-dimensional organization of the yeast nucleus imposes a constraint on such pairing, presumably by dictating the probability with which the two sequences collide with one another.

## Introduction

Genomes have non-random spatial organization: chromosomes, and consequently their encoded genetic elements, are organized into intricate and often dynamic three-dimensional (3D) structures in bacteria [1], fungi [2], [3], insects [4] and mammals [5], [6], [7], [8]. The nature of this organization is important because it can affect basic functions such as chromosome replication and segregation [1], [9], gene expression [4], [7], [10], [11] and the nature of chromosome translocations [6]. Thus, 3D organization adds yet another layer of complexity on top of genetic and epigenetic information. By implication, and as shown by direct experiment, interactions between loci positioned on two different chromosomes, here referred to as *“trans”* interactions, have been shown to be important for a variety of biological processes [7].

Several types of *trans* interactions have been described.

1. Pairing of homologous chromosomes in meiosis. Pairing of homologous chromosomes in meiosis is a case of *trans* interactions in chromosomes [9]. In organisms with the “conventional” meiotic program, such as mammals, plants and fungi, multiple double-stranded breaks in DNA pair homologous chromosomes via homologous recombination [9], [12], [13]. Double-strand break independent pairing precedes this stage [14], [15]. In other organisms, such as *C. elegans* and *Drosophila*, specialized chromosomal sites called pairing centers or pairing sites and their corresponding binding proteins mediate recombination-independent pairing [16], [17], [18], [19], [20].

2. Somatic pairing of homologous chromosomes. In *Drosophila*, homologous chromosomes are paired in somatic cells [4]. Somatic pairing manifests itself in a genetic phenomenon termed transvection, when expression of a gene on one homologous chromosome is influenced by the locus on the other homologous chromosome. For example, an enhancer works *in trans* to activate gene expression (reviewed in [21]. Establishment of somatic pairing occurs in early embryonic development and temporally coincides with beginning of zygotic transcription [22]. Multiple attempts have been made to understand molecular mechanisms responsible for this phenomenon. Two independent screens have been done, and many candidate genes have been identified, but the mechanism of this phenomenon still remains elusive [23], [24], [25]. It has been proposed that the process of zygotic transcription *per se* is what establishes somatic pairing [22], [24]. In both budding and fission yeast, somatic pairing of homologous chromosomes has also been demonstrated [15], [26], [27], [28], [29]. Although somatic pairing is not a genome-wide phenomenon in humans, a case has been described, when somatic pairing of one chromosome's arm and altered gene expression in that region were observed in human cancer cells [30].

3. Transcription factories. In mammalian cell, transcribed genes located on different chromosomes come together in space in the context of transcription factories – nuclear foci that contain proteins necessary for transcription [8], [31]. It has been proposed that there exists a direct correlation between spatial proximity of chromosomal loci in the nucleus and the frequency of chromosomal translocations, commonly observed in human cancers, between those loci [6]. Transcription-induced association of genes located on different chromosomes might contribute to this process [32], [33], [34]. *Trans* interactions have been also been proposed to play a role in regulation of transcription in mouse olfactory system ([35]; but see [36], [37]).

4. Imprinting and monoallelic gene expression. *Trans* interactions between chromosomes might be involved in genetic imprinting – a phenomenon of monoallelic gene expression in mammals, when only one of the alleles (either a maternal one, or a paternal one) of a given gene is expressed, while the other allele is transcriptionally silenced. In mouse, it has been shown that multiple (nonallelic) imprinted loci located on different chromosomes interact in a pair-wise, stochastic manner in embryonic and neonatal tissues [38]. The CTCF protein, which is the major spatial organizer of the mammalian genome [39], seems to be necessary for such interaction [40].

Transient *trans* association of allelic imprinted loci, associated with Prader-Willi syndrome and Angelman syndrome was observed in cells from normal, but not affected individuals [41], but the effect was later attributed to the influence of nucleolus organizer region on three-dimensional organization of the nucleus [42].

5. X-chromosome inactivation. Transient *trans* interaction has been implicated in X-chromosome inactivation – a phenomenon when female placental mammals (such as humans) inactivate one of their two X-chromosomes in a stochastic manner in early embryonic development, such that the female organism is a chimera in which some cells express genes from maternal X-chromosome and others from paternal X-chromosome [43]. The purpose of this event is dosage compensation: by inactivating one of the two X-chromosomes, females express just as much of X-linked genes as males, which have one X-chromosome (and one Y-chromosome). It has been shown that just prior to the initiation of X-chromosome inactivation, presumably at the “counting” and “choice” stages, two XICs (X-inactivation centers) physically come together [43]. Transcription of the relevant elements within the XIC of the X-chromosome, Tsix and Xite, and CTCF protein seem to be necessary for this event [44].

6. DNA replication and repair foci. *Trans* associations have been proposed to occur during DNA replication and repair, as judged by the formation of replication and repair foci in the nucleus of the budding yeast [45], [46], [47].

7. Centromere and telomere clustering in budding yeast. Another example of *trans* interactions in chromosomes is the behavior of centromeres and telomeres in budding yeast. In somatic cells, spindle pole body is embedded in the nuclear envelope and nucleates short interphase microtubules, to which centromeres are attached throughout interphase, leading to centromere clustering, while telomeres are dispersed in several foci anchored on the nuclear envelope [11]. In meiotic cells, celtromere clustering is lost, while telomeres gather in one cluster, resulting in what is known as chromosomal “bouquet” [9], [48].

8. Nuclear periphery provides an opportunity for *trans* interactions. The nuclear periphery seems to be a “special” compartment, where different genetic loci are targeted in a variety of circumstances, and can potentially engage in *trans* interactions with each other. In budding yeast, targeting of genetic loci to the nuclear periphery and/or association with nuclear pores can affect different genes in different ways: it can result in transcriptional silencing [49], transcriptional activation [50], [51], [52], [53], [54], as well as be important for the activity of boundary elements, which stop spreading repressing or activation states of chromatin to adjacent domains [55]. The nuclear periphery has also been proposed to be a specialized compartment where “dangerous DNA elements”, such as telomeres and unrepaired double stranded breaks, gather [56], [57]. In mammalian cells, it seems like nuclear periphery is mostly occupied by heterochromatin, as there are several examples of a gene's relocalization from nuclear periphery to the center of the nucleus, concurrent with transcriptional activation [58], [59], although this does not always seem to be the case [60].

What molecular mechanisms could be responsible for *trans* interactions in chromosomes? In cases that include interaction between two homologous DNA sequences, DNA-DNA homology could play a role and be sensed directly, possibly with involvement of protein “glue”, unusual DNA structures (triplex, G-quartet, Z-DNA) or chromosome-specific “barcode” of simple sequences (reviewed in [15]. Alternatively, and in cases that include interaction between two non-homologous DNA sequences, DNA binding proteins that bind two chromosomal loci and then bind each other could mediate *trans* interactions in chromosomes.

Here, we report and characterize a case of inducible *trans* interaction in chromosomes of the budding yeast, which belongs to the latter category: it occurs between two non-homologous DNA sequences and thus likely relies on a DNA-binding protein.

## Materials and Methods

### Strains

All strains were isogenic haploid or diploid derivatives of *S. cerevisiae* SK1 background ho::LYS2, lys2, leu2, ura3::pGPD1-Gal4(848).ER::URA3. Constructs 1 and 2 were integrated near telomeres XIV and XVI, respectively, unless noted otherwise.

**Figure 1:**
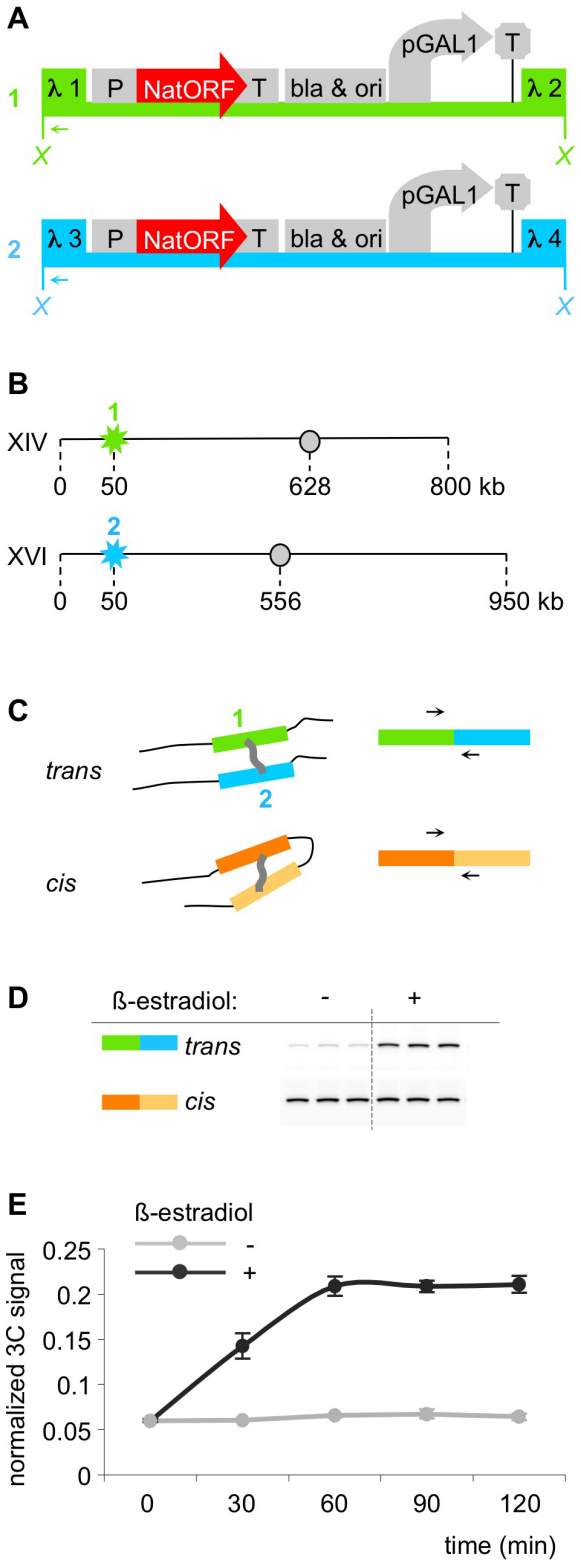
Identification of a system that exhibits inducible *trans* association between chromosomes. A. Map of constructs 1 and 2. Both constructs contain promoter pGal1, transcription terminator tADH1, selectable marker NatMX and bacterial *bla* gene and replication origin, flanked by segments from bacteriophage lambda: construct 1 is flanked by lambda segments 1and 2, construct 2 – by segments 3 and 4. X – XhoI restriction sites used in 3C assay. Arrows – primers used in 3C assay (one primer anneals to lambda 1 segment in construct 1, the second one – to lambda 3 segment in construct 2). B. Schematic representation of genomic integration of the constructs. Construct 1 (flanked with sequences 1 and 2) was integrated near telomere of chromosome XIV. Construct 2 (flanked with sequences 3 and 4) was integrated near telomere of chromosome XVI. Stars – positions of integration; circles – centromeres; numbers indicate kilo base pairs of DNA. C. Schematic representation of the 3C assay. 3C assay relies on chromatin crosslinking and subsequent PCR analysis of ligation junctions between crosslinked segments. *Trans* PCR assays spatial proximity of constructs 1 and 2, while *cis* PCR, which assays spatial proximity between two randomly chosen chromosomal segments, is used for normalization. D. Example of a representative gel showing result of the 3C assay. “−” – culture not treated with β-estradiol, “+” – culture treated with β-estradiol. 3C-PCR using primers that assay constructs 1 and 2 (*trans*) is significantly stronger in the induced compared to uninduced culture, while the normalization PCR (*cis*) is not visibly changed. Each PCR reaction is performed in triplicate. E. Kinetics of induction. Logarithmic culture of yeast was divided into two halves, one half was induced with 1 mkM β-estradiol, and samples from both cultures were taken and crosslinked every 30 minutes. 3C assay was done simultaneously for all crosslinked samples from one experiment. Light grey – uninduced culture, dark grey – induced culture. X-axis – minutes after induction, Y-axis – quantified 3C-signal (see Materials and Methods).

Figure 1. KMY97 – original constructs.

**Figure 2:**
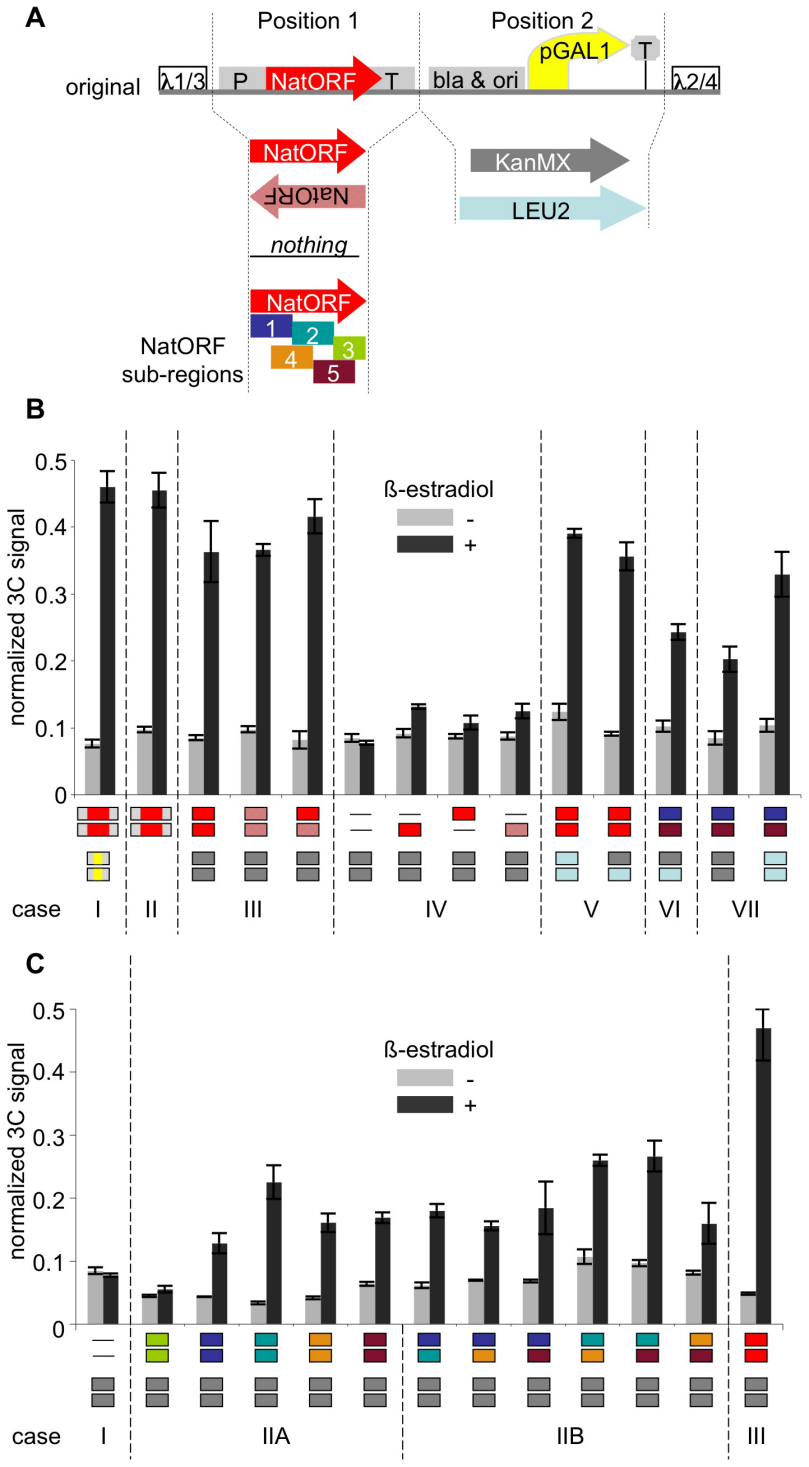
Unique requirements for the inducible *trans* association. A. Map of new constructs in comparison to the original constructs. Position 1 contains either NatMX cassette (Nat ORF flanked by TEF1 promoter and terminator), or Nat ORF in direct or inverted orientation, or one of its five subsequences, or nothing; position 2 contains a selectable marker for transformation: KanMX or LEU2. B. 3C assay in the new constructs. Different combinations of the two constructs were integrated in each strain, at the same loci as shown in Figure 1B. Light grey bars – uninduced cultures; dark grey bars – cultures induced with β-estradiol. Content of positions 1 and 2 is shown below each pair of bars, with color-coding being consistent with Figure 2A. See text for details. C. 3C assay in the new constructs, continued. Different combinations of the two constructs with different sub-sequences of Nat ORF. Light grey bars – uninduced cultures; dark grey bars – induced cultures. Content of positions 1 and 2 is shown below each pair of bars, with color-coding being consistent with Figure 2A. See text for details.

Figure 2B. Case I: KMY97 – original constructs. Case II: KMY165 – NatMX only constructs. Case III: KMY219 – KanMX-NatORF/KanMX-NatORF; KMY222 – KanMX-NatORF-inv/KanMX-NatORF-inv; KMY223 – KanMX-NatORF/KanMX-NatORF-inv. Case IV: KMY218 – KanMX-empty/KanMX-empty; KMY220 – KanMX-empty/KanMX-NatORF; KMY221 – KanMX-NatORF/KanMX-empty; KMY224 – KanMX-empty/KanMX-NatORF-inv. Case V: KMY274 – LEU2-NatORF/LEU2-NatORF; KMY276 – KanMX-NatORF/LEU2-NatORF. Case VI: KMY277 – KanMX-1/LEU2-5. Case VII: KMY266 – KanMX-1/KanMX-5; KMY275 – LEU2-1/LEU2-5.

Figure 2C. Case IIA: KMY259 – KanMX-1/KanMX-1; KMY260 – KanMX-2/KanMX-2; KMY261 – KanMX-3/KanMX-3;KMY262 – KanMX-4/KanMX-4; KMY263 – KanMX-5/KanMX-5. Case IIB: KMY264 – KanMX-1/KanMX-2; KMY265 – KanMX-1/KanMX-4; KMY266 – KanMX-1/KanMX-5; KMY267 – KanMX-2/KanMX-4; KMY268 – KanMX-2/KanMX-5; KMY269 – KanMX-4/KanMX-5.

**Figure 3:**
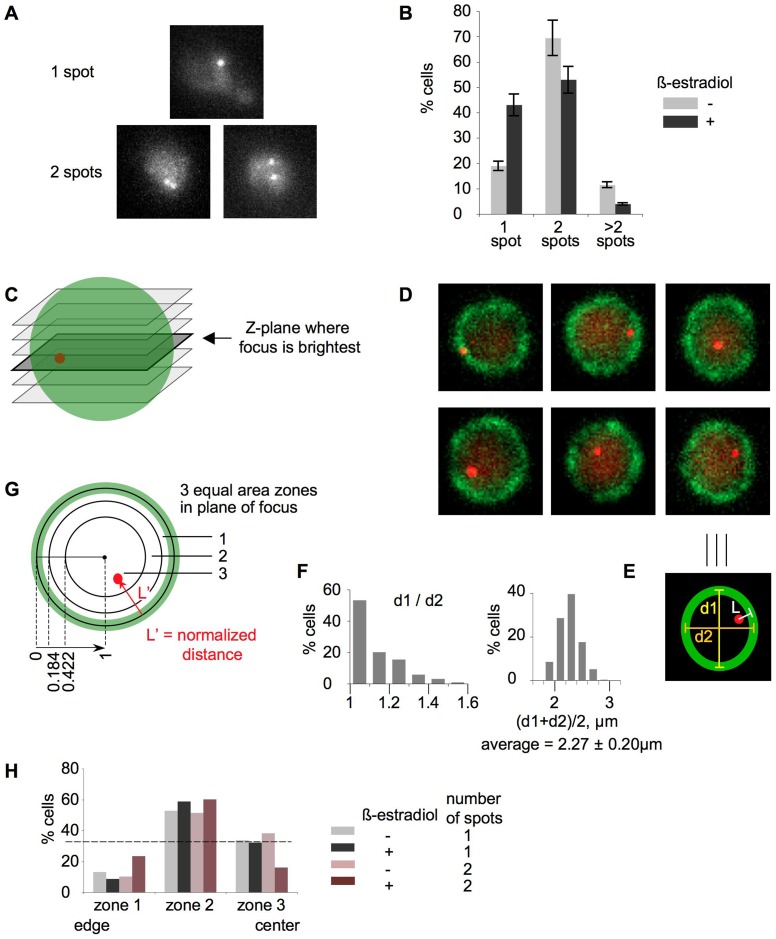
Visualization of inducible *trans* association in 3D in whole cells. A, B – spot counting in fixed cells. A. Representative examples of cells with 1 spot and 2 spots. Cells with one round spot were classified as “1 spot – cells”. Cells with two discernable spots, no matter how close to one another, were classified as “2 spot – cells”. B. Quantitation of the microscopic analysis. Percentages of cells with 1 spot, 2 spots and >2 spots (3 or 4 spots) are plotted for a culture without β-estradiol (light grey bars) and a culture with β-estradiol (dark grey bars). Three independent cultures were analyzed; 500 cells per each sample were scored. C–H. Spot localization analysis in live cells. C. A cartoon showing z-planes. The z-plane with the brightest spot is highlighted. D. Examples of z-planes with the brightest spots. The spots were computationally enhanced for publication purposes. E. Measurements. The definition of the nuclear periphery was based on the perinuclear localization of Nup49-GFP, which manifested itself as a close curve enveloping the nucleus with an average 250 nm thickness (green oval); the true nuclear perimeter was assumed to be centered within this band. In the z-plane with the brightest spot, three parameters were measured: the shortest distance from the center of the spot to the estimated true nuclear periphery (L), long axis of the nucleus (d1) and short axis of the nucleus (d2). The average diameter was calculated by taking the arithmetic mean of the long and short axis. Each spot distance to the nuclear periphery was then normalized to the corresponding average diameter, and divided by two to bring values to the 0–1 scale, where 0 is the periphery and 1 is the center. F. Analysis of nuclear diameters. Left – histogram of d1/d2, where d1 is the longest nuclear axis and d2 is the shortest nuclear axis. Relationship between d1 and d2 is a measure of roundness of nuclei. Right – distribution of average diameters, which were calculated as arithmetic mean of the long and short axis for all categories of cells combined. G. Equal area zones. Normalized distances (L′) were binned into three zones with equal areas. The borders of the zones were defined as follows: 0.184, 0.422, 1 (where 0 is nuclear periphery and 1 is nuclear center). H. Percent of cells with spots in each of the three zones. Four categories of cells were analyzed: 1 spot cells from the culture without β-estradiol (light grey bars); 1 spot cells from the culture with β-estradiol (dark grey bars); 2 spot cells from the culture without β-estradiol (light brown bars); 2 spot cells from the culture with β-estradiol (dark brown bars). Dotted line illustrates the hypothetical scenario on which spots are randomly distributed between the three equal area zones.

Figure 3A–B. KMY208 – case II constructs with *tetO* arrays; leu2::pTetR-GFP::LEU2/leu2.

Figure 3C–H. KMY322 – case II constructs with *tetO* arrays; leu2::pTetR-tdTomato::LEU2/leu2; NUP49-GFP::URA3.

**Figure 4:**
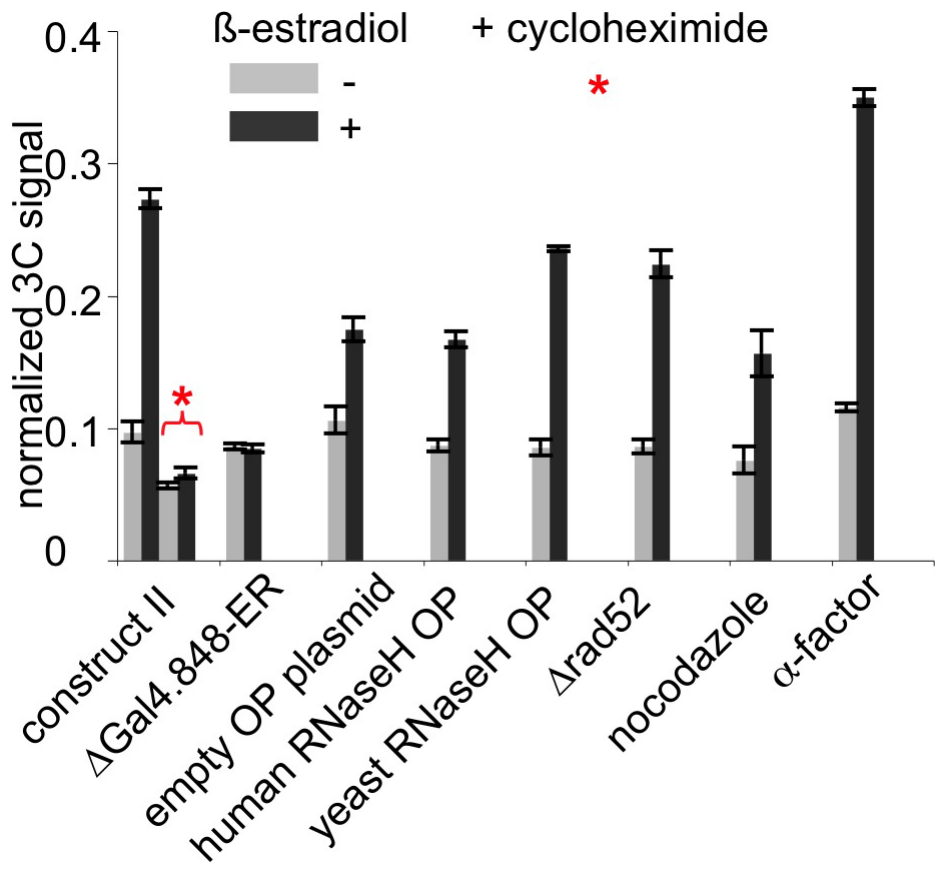
Further characterization of the inducible *trans* association of chromosomal loci. 3C assay in uninduced (light grey bars) and β-estradiol-induced (dark grey bars) cultures, in a constructs shown in Figure 2B, “case II”. Asterisk denotes culture treated with cycloheximide.

Figure 4. KMY97 – case II constructs; KMY135 –ΔGal4(848).ER; KMY225 rad52::LEU2; for RNaseH experiment, KMY97 was transformed with p425-pGPD1-empty, p425-pGPD1-RHaseH1 (yeast) or p425-pGPD1-RNH1 (human).

**Figure 5:**
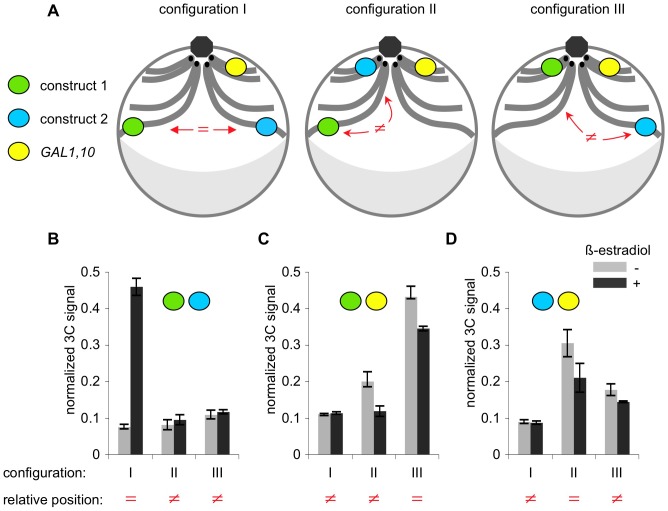
Three-dimensional organization of the genome is important for inducible *trans* association of chromosomal loci. A. Schematic representation of nuclei of the three strains used in this experiment. Constructs 1 and 2 were integrated either 50 kb away from telomere on chromosome XIV and 50 kb away from telomere on chromosome XVI (configuration I), or 50 kb away from telomere on chromosome XIV and HIS4 (configuration II), or at HIS4 and 50 kb away from telomere on chromosome XVI (configuration III), respectively. Green oval – construct 1; blue oval – construct 2; yellow oval – endogenous GAL1,10 locus. GAL1,10 locus is located on chromosome II, about 41 kb away from the centromere II. HIS4 locus is located on chromosome III, about 46 kb away from the centromere III. Integration site 50 kb away from telomere XIV is about 580 away from the centromere XIV. Integration site 50 kb away from telomere XVI is about 500 kb away from the centromere XVI. In vegetatively growing yeast cells, centromeres (black dots) cluster near spindle pole body (grey octagon). Grey crescent denotes nucleolus. Haploid yeast cells contain 16 chromosomes, but only chromosomes II (with GAL1 locus), III (with HIS4 locus), XIV (with construct 1) and XVI (with construct 2) are shown for simplicity. “ = ” – loci positioned at similar latitudes, “≠” – loci positioned at different latitudes. B. 3C analysis of association between constructs 1 and 2 in three configurations. Light grey bars – uninduced cultures, dark grey bars – culture induced with β-estradiol. Increased association between the constructs upon induction is evident in configuration I, but not in configurations II or III. C. 3C analysis of association between construct 1 and endogenous GAL1 locus, in three configurations. No increased association between construct 1 and endogenous GAL1 locus upon induction is observed regardless of configuration. D. 3C analysis of association between construct 2 and endogenous GAL1 locus, in three configurations. No increased association between construct 2 and endogenous GAL1 locus upon induction is observed regardless of configuration.

Figure 5. Original constructs (case I) integrated at different locations: KMY97 – configuration I, construct 1 at telomere XIV, construct 1 at XVI; KMY100 – configuration II, construct 1 at telomere XIV, construct 2 at HIS4; KMY99 – configuration III, construct 1 at HIS4, construct 2 at telomere XVI.

### Construction of plasmids

For construction of original constructs 1 and 2, a linker with multiple restriction sites was inserted into the *Aat*II – *Sap*I portion of pUC18 plasmid, which contains *bla* gene and origin of replication. Into this linker, the following parts were sequentially cloned: NatMX cassette from pAG25 plasmid [61], pGAL1 and T_CYC1_ from pSH47 plasmid [62], 500 bp long fragments of bacteriophage λ protein H (tail component) gene, called lambda 1,2,3 and 4 (1 and 3 flank construct 1; 2 and 4 flank construct 2) and 400 bp long fragments for genomic integration into the target sites.

For construction of new constructs 1 and 2, the plasmids were re-arranged such pGAL1 and T_CYC1_ were deleted, and bacterial parts were left out upon digestion prior to yeast transformation. Nat ORF sub-regions were amplified using PCR. NatMX cassette, KanMX cassette from pYM13 plasmid [63], or LEU2 cassette from YIplac128 plasmid were used for selection in yeast transformation.

### Chemical treatments

β-estradiol induction: 5 ml YPD was inoculated with a single colony of yeast and grown overnight at 30°C, after which 1 ml of the overnight culture was transferred into 100 ml YPD and grown for 4 hours. 50 ml of the culture was transferred to a new flask, and 5 µl of 10 mM stock solution in of β-estradiol (Sigma E2758) in ethanol was added to the new flask (1 µM final concentration), while the first flask was left untreated. Both flasks were incubated for additional 90 min at 30°C, after which samples of cells were taken for 3C or microscopy (final OD_600_∼1.0).

Cycloheximide: cells were grown as above; after the cultures were split, 100 µl of 100 mg/ml cycloheximide solution in DMSO was added to both uninduced and induced cultures (200 µg/ml final concentration), simultaneously with β-estradiol induction.

Nocodazole arrest: 5 ml YPD was inoculated with a single colony of yeast and grown overnight at 30°C, after which 1 ml of the overnight culture was transferred into 100 ml YPD and grown for 4 hours. 150 µl of 10 mg/ml nocodazole (Sigma M1404) solution in DMSO was added (15 µg/ml final concentration). After 1.5 h, the culture was spit in half and one half induced with β-estradiol exactly as above. Microsopy was used to confirm G2/M arrest (large budded cells with DAPI-stained body at the neck) both before and after induction.

α-factor arrest: 5 ml YPD was inoculated with a single colony of yeast and grown overnight at 30°C, after which 1 ml of the overnight culture was transferred into 100 ml YPD pH 3.9 and grown for 4 hours. Add 50 µl of 10 mM alpha-factor (Zymo research Y1001) (5 µM final concentration). After 1.5 h, the culture was spit in half and one half induced with β-estradiol exactly as above. Microsopy was used to confirm G1 arrest (unbudded cells with mating projections) both before and after induction.

### 3C assay

10 mL aliquot of exponentially growing yeast culture (OD600∼1) was treated with 270 µL of 37% formaldehyde (1% final concentration) for 15 min at room temperature, reaction was quenched with 1080 µL of 1.25M glycine for 5 min at room temperature, after which cells were pelleted by centrifugation, **r**esuspended in 250 µL FA-lysis buffer (50 mM Hepes-KOH pH 7.5, 140 mM NaCl, 1 mM EDTA, 1% Triton X-100, 0.1% sodium deoxycholate), transferred into a 2 ml tube with 0.5 g of acid washed glass beads and kept on ice. Cells were broken by vigorous vortexing (1 min vortex followed by 1 min on ice, repeated 7 times). Another 250 µL FA-lysis buffer was added to cells and after vortexing, all liquid was transferred to a new tube kept on ice. The beads were washed with 500 µL FA-lysis buffer, and after vortexing, all liquid was transferred to the same tube. The last step was repeated, so the total volume of obtained lysate was 1.5 ml. The lysate was centrifuged for 15 min at 14,000 rpm at +4°C in a microcentrifuge, the supernatant was discarded and the crosslinked chromatin in the pellet was resuspended in 300 µL H20, 50 µL 10× NEB2 restriction buffer and 50 µL 1% SDS and heated for 30 min at 65°C, after which 50 µL 10% triton X-100 was added.

For restriction digest, 600 U of XhoI (New England Biolabs) was added and reactions were incubated overnight at 37°C. Restriction enzyme was inactivated by addition of 55 µL of 1M Tris-HCl pH = 8.0 and 100 µL of 10% SDS, and heating for 20 min at 65°C.

For ligation, 7.125 mL of H_2_0, 1 mL of 10% triton X-100, 1 mlL of 10× ligation buffer, 100 µL of 100 mM ATP, 100 µL of 10 mg/ml BSA and 20 U of T4 DNA ligase (Invitrogen) was added, and reactions were incubated at room temperature for 2 hours.

Crosslinks were reversed by adding 200 µL of 0.5M EDTA, 330 µL of 1.5M Tris-base, 390 µL of H_2_O, 480 µL of 5M NaCl, 600 µL of 10% SDS and 50 µL of 20 mg/mL proteinase K and incubation for 4 hours at 65°C.

DNA was precipitated by adding 30 ml of ethanol and centrifuging in ss-34 rotor (Sorvall) at 12,000 rpm for 20 min, dissolved in 400 µL TE and extracted with phenol-chloroform three times, after which precipitated again by adding add 40 µL 3M NaAc pH 5.2 and 1 mL EtOH, and centrifuging for 10 min on maximum speed in a tabletop centrifuge. DNA was washed 3 times with 70% ethanol, dried for 5 min in speed-vac, dissolved in 200 µL TE and treated with 2 µL of 10 mg/mL RNase A for 30 min at 37°C, after which it was heated for 20 min at 65°C.

2 µL of 3C template was used for each PCR reaction. For each template, 6 PCR reactions using Phusion Hot Start Flex polymerase were performed: with *trans* and *cis* pairs of primers, each one in triplicate. *Trans* primers were KM-L1b-2: AGGGTTGAGTTGCCCTGATACC and KM-L3b-2: ATTTGCTCCGGCATGCTTC, *cis* primers were KM4a: ACACTATCAGACCCTACAGTTAAGGAGAAA and KM9a-2: AAGCAAATGGCGTCCAAAATGTTCGACTTA. *GAL1* locus primer was Gal1-L: GAAAACCTGCTCTTACTGGATGCTGAC. PCR program was 98°C 1 min, [98°C 10 sec, 68°C 45 sec, 72°C 45 sec] 30 times, 72°C 5 min. Linear mode of amplification around 30 cycles was verified for both *trans* and *cis* pairs of primers. 15 µl of PCR reaction was loaded on 1.5% TBE-agarose gel with 0.5 µg/ml ethidium bromide and after electrophoresis, intensity of bands was quantified using BioDar Molecular Imager FX phosphorimager using Quantity One software (BioRad). For each 3C template, the intensity of *trans* signal was normalized to the intensity of *cis* signal and averaged among the triplicates.

### Microscopy

*S. cerevisiae* SK1 haploid cells are extremely clumpy and therefore are challenging objects for microscopic analysis. Diploid cells, in contrast, are less clumpy. Thus, all microscopy was carried out using diploid strains (KMY208; KMY322 above). 3C analysis confirmed that pairing is as robust in diploid strains as in haploid strains (data not shown).

Microscopy was performed on an inverted microscope (Nikon Ti-e) with a 100× oil immersion objective (NA 1.45 lambda), and illuminated by a 6 channel LED system (Lumencore). The camera used was an EM-CCD (Hamamatsu ImagEM 512) with a pixel dimension of 16 microns, coupled to a 2.5× tube magnifier. 3D z-stacks were taken with a piezo stage (Prior Nanoscan-Z 100) at 250 nm intervals for a total of 44 z-steps with a 32 ms exposure per slice. Further image processing was done using Matlab (Mathworks) and ImageJ (NIH). For Figure 3A–B, cells were fixed in 40% Ethanol/0.1M sorbitol. For Figure 3C–G, cells were imaged live. For Figure 3C–G, the two emission channels of eGFP and tdTomato were acquired one complete z-stack after another with filter sets 49002 (Chroma) and LF488/561 (Semrock) respectively.

Spot to nuclear periphery distance followed convention as outlined in [64], [65]. In summary: given a 3D stack from the tdTomato channel, the diffraction limited spots were computationally enhanced by taking the normalized cross-correlation with a gaussian kernel. The z-slice that contained the brightest z profile of the diffraction limited spot was then selected, and its centroid in xy was scored as its position. The definition of the nuclear periphery was based on the perinuclear localization of Nup49-GFP, which manifested itself as a closed curve enveloping the nucleus with an average 250 nm thickness; the true nuclear perimeter was assumed to be centered within this band. Given the selected z-slice, three parameters were measured using the line tool in ImageJ: 1) The shortest line segment from the centroid of the spot to the estimated true nuclear periphery (L), 2) the long axis of the nucleus (d1), 3) the short axis of the nucleus (d2). The average diameter was calculated by taking the arithmetic mean of the long and short axes. Each spot distance to the nuclear periphery was then normalized to the corresponding average diameter, and divided by two. This operation brings spot-periphery distance values to a 0–1 scale, where 0 is the periphery and 1 is the center of the nucleus. These normalized distances (L′) were then binned into three zones with equal areas as in [64], [65]. The borders of the zones were defined as follows: 0–0.184, 0.184–0.422, 0.422–1 (where 0 is nuclear periphery and 1 is nucleus center). Any z-slice that did not have a clear 250 nm thick band of Nup49-GFP was ignored. This filtering primarily eliminated z-slices located at the top and bottom of the nucleus.

## Results and Discussion

### Identification of a system that exhibits inducible *trans* association between chromosomes

We were interested to study recombination-independent homology-dependent *trans* association between chromosomes, and the possible role of transcription in that process. To this end, we constructed a pair of tester constructs in which the existence and requirements for such a process could be tested and where spatial juxtaposition could be analyzed by chromosome conformation capture (“3C”), [66]. Identical cores in the two constructs were flanked by different pairs of sequences as required for 3C analysis (lambda 1,2 and lambda 3,4, respectively). The identical cores included an inducible promoter *pGAL1* and an expressed *NatMX* selectable marker (nourseothricin resistance, [61], among other determinants (Figure 1A). The two constructs were integrated ectopically in two different chromosomes of a haploid strain. Specifically, the constructs were integrated sub-telomerically at the long arms of chromosomes XIV and XVI (Figure 1B). This location was chosen because of the possibility nuclear envelope association of telomeres might promote association of the two constructs by reducing the dimensionality of the search process. The long arms of chromosomes XIV and XVI lack the subtelomeric homology shared by some chromosome's arms, so any potential complication from the homology-driven interactions of telomeres could be avoided.

For 3C analysis (Figure 1C), cell samples were subjected to formaldehyde crosslinking, restriction digestion, dilution to reduce the relative concentrations of non-crosslinked segments, and ligation of created ends. Crosslinks were then reversed and ligation junctions assayed by PCR. Spatial proximity of the two constructs in the cell nucleus at the time of crosslinking is reflected in a higher probability that the corresponding digestion-produced ends will be ligated to one another, resulting in a higher level of PCR signal. Thus, if the two tester constructs, initially present in *trans* on different chromosomes, come together in space, the level of the diagnostic PCR signal will correspondingly increase. Parallel *cis* reactions were also carried out using the same samples, for a pair of segments located nearby (on the same chromosome). These reactions control for variations in samples, unrelated to changes in spatial proximity. PCR product levels for *trans* tester constructs were normalized to the level of the PCR product for the *cis* tester construct in the same experiment. Increased spatial juxtaposition of the two tester constructs was thus revealed by an increase in the ratio of the PCR signals in the *trans* versus *cis* cases, referred to below as the “normalized 3C signal”. A typical gel is shown (Figure 1D).

Transcription from the *pGAL1* promoter in the constructs was induced with β-estradiol in a strain expressing *GAL4(848).ER* [67], [68]. *GAL4(848).ER* is *GAL4* transcription activator truncated at amino acid 848 such that it no longer interacts with, and is no longer inhibited by, *GAL80*. Instead, hormone-binding domain of a vertebrate estrogen receptor is fused to its C-terminus, providing an opportunity for rapid induction. Upon addition of β-estradiol to an exponentially growing culture, the normalized 3C ratio for the tester construct increased progressively over time for a period of an hour, after which it reached plateau (Figure 1E). No increase was observed in the absence of β-estradiol. Thus, the created constructs, located in *trans* on two different chromosomes, exhibit β-estradiol-induced association.

### Unique requirements for the inducible *trans* association

Since the effect that we observed reached plateau at 60 minutes, all subsequent experiments were done at 90 minutes post induction (to allow for culture variability). To determine which genetic determinants of the tester constructs were required for their association, derivatives of the original constructs were created and examined in several combinations which tested the roles of the *pGAL1* promoter, transcription terminators inserted downstream of the region transcribed by that promoter, the nature of the transcribed region and the selectable marker, and the extent to which the two testers shared common (homologous) sequences (Figure 2A). Surprisingly, association occurs, specifically upon treatment with β-estradiol, in a pair of constructs that contain neither a *pGAL1* promoter in *cis* nor any DNA sequence homology of any kind, where the only requirement for association is the presence of two subsequences of the *Nat* gene which need not be overlapping in sequence, on both constructs. The following data supports this conclusion.

Derivatives of the original constructs included different combinations of elements at positions 1 and 2 (Figure 2A): position 1 contained either *NatMX* cassette (*Nat* ORF [open reading frame] flanked by *TEF1* promoter and terminator), or *Nat* ORF in direct or inverted orientation, or one of its five subsequences, or nothing; position 2 contained a selectable marker for transformation (*KanMX* or *LEU2*). None of the derivatives contained the *pGAL1* promoter.

Figure 2B shows compares the 3C-defined levels of association for the original construct pair (case I) with those for pairs of constructs carrying different combinations of information at positions 1 and 2 (cases II–VII). The following conclusions emerge:

- Deletion of *pGAL1* (along with the rest of the content at position 2 in the original constructs) did not diminish the association between the two constructs (case II versus case I). Thus, the presence of *pGAL1* within the tester constructs is not relevant. More specifically: it is not the induction of transcription in *cis* that is causing the association of the constructs.
- Constructs that contained only *Nat* ORF at position 1 (in either orientation) and the *KanMX* marker at position 2 (case III) display as high level of association upon addition of β-estradiol as the original constructs (case I). These case III constructs lack all of the determinants present in the original constructs except *Nat* ORF; thus, all of these other determinants are dispensable.
- If, in addition to deletion of all other original determinants, *Nat* ORF is present on only one of the two tester constructs, pairing is not observed above background (case IV). Thus, in comparison with case III, this result implies that *Nat* ORF must be present on both constructs for pairing to occur. Furthermore, if both constructs carry *Nat* ORF, homology at position 2 (marker) is dispensable, as constructs with different markers also exhibit high level of association upon induction (case V).
- The requirement for *Nat* ORF to be present on both constructs might reflect a requirement for overall DNA/DNA homology. However, unexpectedly, this is not the case: β-estradiol-induced pairing is observed if the two tester constructs carry non-overlapping sub-sequences of the *Nat* ORF, although the association level is somewhat reduced (case VI). Importantly, these constructs also have different markers at position 2, so there is no homology between any parts of the two constructs. Moreover, if the two constructs carry identical marker sequences at position 2, there is no major change in the level of pairing (case VII).

*Nat* ORF is 573 bp long. This segment was divided into five partially-overlapping subregions of this sequence, each about 200 bp long (Figure 2A). The abilities of these subregions to promote pairing was then examined with the each of the regions present in either homozygous configuration (i.e. with the same subregion on both tester constructs) or in various combinations of heterozygous configurations (i.e. with different subregions on the two testers) (Figure 2C). Two conclusions emerge: (i) Four of the five subregions are sufficient to promote pairing when present in homozygous form; sub-sequence 3 is the only one that fails confer pairing (case IIA; compare with case I). (ii) The four regions that can promote pairing in homozygous form also promote pairing in all heterozygous combinations (case IIB compare with case I). In all cases, the level of pairing somewhat lower than that of the full-length *Nat* ORF (case III).

### Visualization of inducible *trans* association in 3D in whole cells

To confirm the existence of β-estradiol-induced pairing as defined by 3C, and to further explore the positions of paired loci within the 3D volume of the cell, we directly visualized the pairing of tagged loci by fluorescent repressor/operator arrays [69], [70], [71]. 1 kb arrays comprising 30 *tet0* binding sites were introduced into the pair of tester constructs described in Figure 2B, “case II”, and visualized by expressing fluorescently-tagged tetracycline repressor (TetR-GFP; [70]. The arrays are shorter than those used in previous budding yeast studies [70], [71], [72], [73] in order to minimize the possibility that the bulkiness of the repressor/operator array would decrease the mobility of the corresponding loci. The positions of the two spots were monitored in 3D (by taking z-stacks of whole cells and generating a maximum brightness projection) over time after β-estradiol-mediated induction and, as a control, in the absence of induction. The same results were observed in both fixed cells (Figure 3AB) and live cells (not shown).

Uninduced and β-estradiol-induced cultures both contain some cells with one spot and some cells with two spots (Figure 3A), and a minor fraction of cells with 3 or 4 spots. The latter probably correspond to cells in which the constructs have been replicated and sister chromatids are undergoing segregation. Additionally, after estradiol addition, the percentage of one-spot cells in the induced culture increases over time, not only in absolute terms, but relative to that in an uninduced culture analyzed in parallel (Figure 3B). The magnitude of the increase was somewhat smaller than that observed with 3C (e.g. Figure 2). This difference may reflect the fact that detection of pairing by 3C requires closer juxtaposition than cytological detection of a single paired signal, and therefore background signal in 3C might be lower than that of microscopy.

We also asked whether the loci that paired after β-estradiol induction were present preferentially on the nuclear periphery. This is of interest because of multiple known functional roles of localization to the nuclear periphery (Introduction); because the loci being tested were positioned near their respective telomeres, which can tend to be peripherally-associated [11]; and because, in particular, transcription-induced re-localization of galactose-inducible genes has been demonstrated in yeast [52].

Disposition of constructs relative to the nuclear periphery was determined in a strain where the same constructs as those used in Figure 3AB (Figure 2B, “case II” constructs marked with 1 kb arrays comprising 30 *tet0* binding sites) were visualized via TetR-tdTomato (red) and nuclear envelope was marked by NUP49-GFP (green, [64], [65]). Live cells from uninduced and β-estradiol-induced cultures were imaged by taking z-slices. Then, for each cell, the slice(s) with the brightest spot was selected (Figure 3C). Examples of such z-slices are shown in Figure 3D. In each such slice, three parameters were measured (Figure 4E): the shortest distance from the center of the spot to the estimated true nuclear periphery (L), longest axis of the nucleus (d1) and perpendicular short axis of the nucleus (d2). The average diameter was calculated by taking the arithmetic mean of the long and short axes. Analysis of d1 and d2 values revealed that within the z-slices selected for analysis, nuclei were fairly round (d1/d1≤1.1) and fairly similar in size (Figure 4F). Each spot distance to the nuclear periphery was then normalized to the corresponding average diameter, and divided by two, thus bringing values to a 0–1 scale, where 0 is the periphery and 1 is the center. The L′ values of the analyzed slices (one per nucleus) were then binned into three categories whose borders define three zones of equal area (Figure 4G). These borders corresponded to normalizes distances (L′) as follows: L′<0.184, 0.184<L′<0.422, 0.422<L′<1, where 0 is nuclear periphery and 1 is the nuclear center (Figure 4G). These bins were chosen in accord with previous studies ([64], [65]) for the following reason. If spots were randomly distributed throughout the nucleus, they should occur with equal frequency in each of the three zones. On the other hand, if spots occurred preferentially at/close to the nuclear periphery or away from the periphery, a corresponding bias in the distribution of spots to the different zones should be observed. Such distributions were defined for four categories of cells: (i) uninduced cells with 1 spot; (ii) induced cells with 1 spot; (iii) uninduced cells with 2 spots; and (iv) induced cells with 2 spots). In all four cases, spots did not localize preferentially to the nuclear periphery and, instead, occur preferentially in the middle zone, away from both the periphery and the center of the nucleus (Figure 4E).

These results show that loci that have become paired after β-estradiol induction do not re-localize to the nuclear periphery. More generally, there is no obvious difference in the disposition of the tester constructs in the presence or absence of β-estradiol induction, regardless of whether the two constructs colocalize or not.

### Further characterization of the inducible *trans* association of chromosomal loci

What could be responsible for the inducible *trans* association of chromosomes that we observe? Addition of β-estradiol activates *GAL4(848).ER* with the result that transcription of *GAL* genes is strongly induced, but some other genes (not related to galactose metabolism) are induced as well [74]. However, we have established that our constructs undergo association upon addition of β-estradiol even when they don't contain *pGAL1* promoter. Thus, pairing is not induced as a result of the induction of transcription *in cis*. The straightforward alternative possibility would be that β-estradiol mediated induction of transcription elsewhere in the genome results in the expression of a protein “X” which, in turn, mediates pairing (*e.g.* by binding of the protein to sequences common to the non-overlapping DNA regions that can engage in pairing; below). To test this hypothesis, we looked at whether protein synthesis *de novo* was required, by treating cells with a protein synthesis inhibitor, cycloheximide, simultaneously with addition of β-estradiol. We found that addition of cycloheximide completely abolished induction of association between the constructs (Figure 4).

Another possibility could be that β-estradiol mediates changes that are somehow unrelated to its action upon *GAL4(848).ER*. This possibility was excluded by analyzing pairing in strains that specifically lacked *GAL4(848).ER*: elimination of *GAL4(848).ER* completely abolishes induction of association between the constructs (Figure 4).

We also tested for the roles of several other factors that could potentially be involved in estradiol-induced pairing using the constructs shown in Figure 2B, “case II”.

(1) Transcription-caused R-loops. Overexpression of RNaseH, which is known to eliminate R-loops [75] had no effect on pairing (Figure 4). (2) Recombination. Elimination of Rad52, which is essential for all types of homologous recombination and recombinational repair in yeast, also had no effect (Figure 4). (3) Cells arrested in G1 (with α-factor) had a slightly higher level of association than did cells in an asynchronous culture. (4) Oppositely, G2-arrested cells had a slightly lower level of pairing than an asynchronous culture (Figure 4).

### Three-dimensional organization of the genome is important for inducible *trans* association of chromosomal loci

The genome of vegetatively growing yeast cells has a highly organized three-dimensional structure: centromeres cluster on one pole of the nucleus, adjacent to the spindle pole body, while telomeres are dispersed and cluster in multiple foci associated with the nuclear envelope [11]. Thus, the physical distance of a given locus from the centromere and consequently from the centromere clustering pole of the nucleus is therefore, on average, roughly proportional to its genomic distance from the centromere [2], [3]. The yeast nucleus might thereby be subdivided into sections at different “latitudes”, with loci located at similar genomic distances from their corresponding centromeres occupying similar latitude sections. Spatial organization will necessarily influence the probability with which two given loci will come in spatial proximity with one another, such that two loci located at similar distances from their centromeres on average will be located closer to one another than those loci that are located at very different distances from their centromeres (*e.g.* [29]. This effect could, in turn, influence the probability that two loci will collide and, in our system become stably paired.

To investigate this possibility, we constructed three strains in which the original versions of constructs 1 and 2 (from Figure 1) were integrated at centromere-proximal and telomere-proximal positions on different chromosomes such that they were either present at similar predicted latitudes (with both constructs at telomere-proximal positions; Figure 5A blue and green circles in configuration I) or very different latitudes (with one construct centromere-proximal and one construct telomere-proximal; Figure 5A, blue and green circles in configurations II and III). 3C analysis shows that significant pairing is observed only for the same-latitude case (III) and not for the different-latitude cases (I, II) (Figure 5B). We also tested two more “identical latitude” scenarios, where the two constructs were placed at allelic loci in diploid cells (sub-telomere XIV/sub-telomere XIV, sub-telomere XVI/sub-telomere XVI) and the results were identical to that in the sub-telomere XIV/sub-telomere XVI configuration (data not shown).

We were concomitantly interested to know the dispositions of the construct-marked loci with the endogenous *GAL1* locus, in light of the fact that transcription-induced re-localization of galactose induced genes is such a prominent feature in yeast biology [50], [51], [52], [53], [54]. Analysis of these dispositions in the same experiments used for analysis of inter-construct pairing revealed no significant increase in association with the endogenous *GAL1* locus upon β-estradiol induction for any of the constructs (Figure 5CD). However: the *GAL1* locus is centromere-proximal, and 3C signals are highest for interaction of *GAL1* with either construct when present in a centromere-proximal position than for either construct when present in a telomere-proximal position (Figure 5CD). These findings further support the existence of the polarized 3D organization inferred from previous studies (above).

### Model

**Figure 6:**
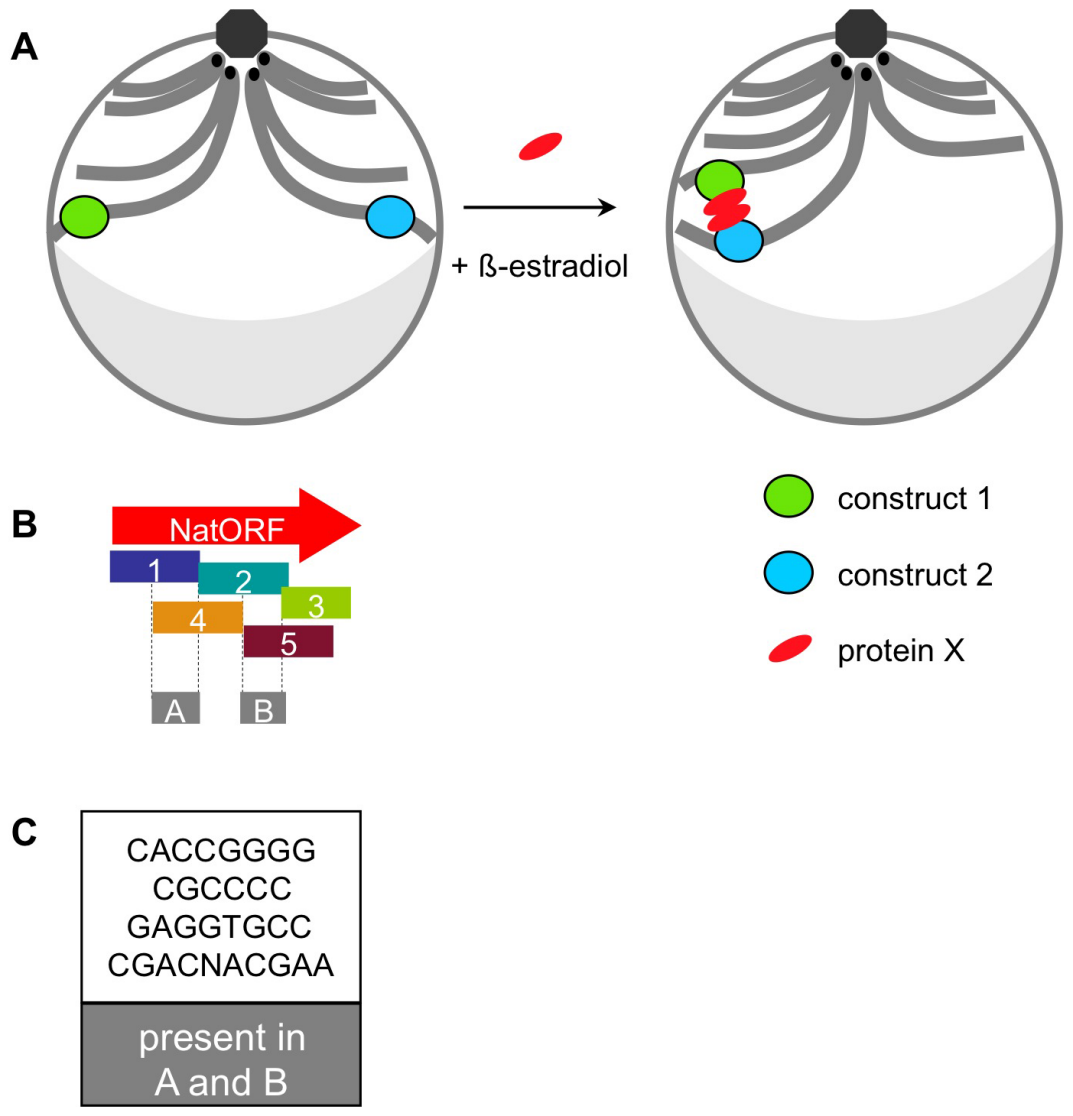
Model. A. Addition of β-estradiol induces expression of protein “X”, which binds to constructs 1 and 2 and pairs them. Schematic representation of a yeast nucleus before and after induction: green oval – construct 1; blue oval – construct 2; red oval – protein “X”; centromeres – black dots; octagon – spindle pole body; grey crescent – nucleolus. B. Five sub-sequences of the Nat ORF. Box A – overlapping region between sub-sequences 1 and 4. Box B – overlapping region between sub-sequences 2 and 5. C. Short DNA sequences contained within both box A and box B.

We show above that two DNA constructs, inserted in chromosomes of the budding yeast in *trans*, undergo spatial association if the following requirements are met: (1) cells are treated with β-estradiol, (2) *GAL4(848).ER* transcription factor is present, (3) protein synthesis *de novo* is occurring, (4) the constructs are located at similar “latitudes” in the nucleus and (5) both constructs contain one of the subsequences of the *Nat* ORF (which need not share overlapping sequence). While other explanations for this phenomenon are possible, the following model seems readily consistent with the data. Addition of β-estradiol activates *GAL4(848).ER* transcription factor, which induces expression of a protein “X”. Protein X binds *Nat* ORF sub-sequences in constructs 1 and 2 and the two bound protein X's then bind each other, pairing their underlying DNAs (Figure 6A).

In the context of this hypothesis, potential binding sites for protein X can be identified. Sub-sequences 1,2,4 and 5 (but not 3), in all possible combinations, were competent to promote pairing (Figure 2C). If this pairing is mediated by protein X, there must be a cognate binding site present within all four sub-sequences. Sub-sequences 1 and 4 share some sequence overlap, as do sub-sequences 2 and 5 (Figure 6B, box A and box B); however, there is no overlap between box A and box B. Thus, there should be a putative protein X binding site present in each of the two shared regions. We have identified several short DNA sequences which meet this critereon: they present in both box A and box B and therefore in all of the sub-sequences 1,2,4 and 5 (Figure 6C).

Pairing of tester constructs is dependent on three dimensional architecture of the genome with loci located in the same “latitude” being permissive and those located at different “latitudes” being restrictive. These findings support previous observations regarding yeast 3D chromosome disposition and are consistent with the simple idea that spatial proximity promotes pairing by increasing the frequency with which the two loci randomly collide with one another.

We further find that G1-arrested cells show an increased level of association, while G2/M-arrested cells show a decreased level of association (Figure 4). Interestingly, the same effects were observed for somatic pairing in budding yeast as analyzed by FISH [27]. These effects could reflect differences in genome organization or chromosome state. Alternatively, they could be explained by the *pair-wise* nature of interaction between the two constructs. Specifically: In G1 phase, both constructs 1 and 2 each exist in one copy in the nucleus, and construct 1 can only engage in a pair-wise interaction with construct 2. In G2, however, each construct is replicated and therefore is present in two copies, on two sister chromatids. Sister constructs could act as competitors, such that sister construct 1 would interact with the other sister construct 1 (and sister construct 2 would interact with the other sister construct 2) preventing interactions between construct 1 and construct 2. Given that 3C only detects interactions between construct 1 and construct 2, but not between sister constructs (1 and 1, or 2 and 2), such model would result in a higher detected level of interaction in G1, a lower level in G2 and an intermediate level in an asynchronous culture, exactly as observed.

We tried two approaches to identify protein X. First, we searched for proteins, which could potentially bind both sequences A and B. Mig1 emerged as a possible candidate. However, the two constructs still paired in Mig1 knockout (data not shown). We also looked at possible candidate proteins among those induced by the GAL4-ER system [74]. We knocked out one such candidate, a protein encoded by *yel057c* locus, but pairing of the two constructs still persisted in that knockout strain.

Alternative explanations could exist. Since the constructs used to examine pairing are bacterial sequences, they might not support normal chromatin assembly, which in turn might trigger increased mobility and thus indirectly promote interaction of the two constructs. Correspondingly, cycloheximide might affect chromosome mobility, thus preventing the two constructs from finding one another.

DNA-binding proteins have been implicated in *trans* interactions in chromosomes in multiple studies [16], [17], [19], [39], [40], [43]. It would be interesting to determine the identity of protein X, which could be responsible for the effect that we observed.
